# The Effects of Functional Training on the Ambulatory Blood Pressure and Physical Fitness of Resistant Hypertensive Elderly People: A Randomized Clinical Rehearsal with Preliminary Results

**DOI:** 10.3390/ijerph21081015

**Published:** 2024-08-01

**Authors:** Jenifer Kelly Pinheiro, Marcos Antonio Araújo Bezerra, Bárbara Raquel Souza Santos, Antônio Gomes de Resende-Neto, Rogério Brandão Wichi

**Affiliations:** 1 Department of Physical Education, Federal University of Sergipe, Aracaju 49060-108, SE, Brazil; jenifer@leaosampaio.edu.br (J.K.P.); barbararaquel24@academico.ufs.br (B.R.S.S.); neto.resende-edf@hotmail.com (A.G.d.R.-N.); 2 Centro Universitário Dr. Leão Sampaio, Juazeiro do Norte 63041-140, CE, Brazil; marcosantonio@leaosampaio.edu.br

**Keywords:** systemic arterial hypertension, elderly, physical exercise

## Abstract

Objective: This study evaluated the impact of functional training (FT) on the ambulatory blood pressure and physical fitness of resistant hypertensive older adults. Method: This randomized clinical and controlled rehearsal involved 15 participants from Juazeiro do Norte-CE divided into two groups: a control group (CG), *n* = 7, without physical training, and an experimental group (EG), *n* = 8, subjected to 24 sessions of FT. The comparative analysis included ambulatory blood pressure (24 h mapping) and physical fitness (Senior Fitness Test), using an ANOVA of two factors, an alpha of 0.05, and a post hoc by Bonferroni where necessary. The dimension of the intervention effect was verified using Eta Squared. Results: The results show that FT promoted a significant reduction in systolic blood pressure (SAP) during the day and at night, as well as improving physical fitness, including the force/resistance of the lower and upper limbs, physical mobility, and aerobic resistance. The flexibility of the upper and lower limbs was not affected. Conclusion: It is concluded that FT is effective in reducing SAP and improving physical fitness in resistant older adults. However, this type of training may positively influence general physical fitness of older adults with resistant hypertension.

## 1. Introduction

Aging is considered a progressive process that occurs during life and changes all the systems of an individual, resulting in alterations of physiological patterns mutually with biopsychosocial factors [1]. According to the World Health Organization, between 2015 and 2050, the population of people aged over 60 years will almost double, increasing from 12% to 22% [2]. In Brazil, it is estimated that there will be around 41.6 million older adults by 2030, and the population proportion until 2060 may comprise a third of the total population [3].

In the face of this increase, it also becomes crucial to consider the health conditions of this population, considering that this expansion in life expectancy is inversely proportional to the attainment of improved life quality. Thus, increased age increases the likelihood of the negative aspects of aging, such as the emergence of chronic degenerative diseases and the mental fragility of senile individuals [4]. In this respect, aging is considered a risk factor for cardiovascular disease appearance [5] and functional decrease [6].

According to the cardiovascular prevention guidelines of the Brazilian Cardiology Society [7], cardiovascular diseases are the primary diseases responsible for the high number of deaths in the world and Brazil, and they are considered a public health problem. It is estimated that, in 2019, around 17.9 million people in the world died due to cardiovascular diseases, representing 32% of total deaths [8]. In Brazil, the values reached 27.3% of deaths in 2017 [9].

Among cardiovascular diseases, systemic arterial hypertension (SAH) is highlighted. It is considered a multifactorial disease, characterized by an increase in and sustenance of pressure levels greater than or equal to 140 mmHg of systolic pressure and greater than or equal to 90 mmHg of diastolic pressure, consistently causing damage to the arteries, heart, and other organs such as the brain and kidneys [10]. In addition, the prevalence of SAH increases with age, increasing to over 60% in older adults [11]. Moreover, among the hypertensive population, it is estimated that around 10% to 20% suffer from resistant hypertensive (RH) conditions [12]. An RH person is considered to be someone who maintains elevated blood pressure levels even when taking three or more antihypertensive medicines, including beta-blockers, or patients whose blood pressure is controlled with four or more antihypertensive cases [13]. In this regard, these patients lack pharmacological responsivity [14]; in other words, they do not achieve the objectives of administered therapies, even by using medicines in combined doses.

In the face of the difficulties of controlling blood pressure (BP) in resistant patients, new methods of treatment have been explored in the literature, for instance, the use of invasive devices related to sympathetic inhibitor interventions; however, they need more confirmation before they can be considered broadly applicable [15]. Physical exercise is indicated as a non-pharmacological strategy for the control and prevention of resistant hypertension, being considered a first-line approach for treatment [14].

It is essential to point out that there is a prevalence of hypertensive cases with functional limits [16], highlighting the importance of interventions related to minimizing the effect of hypertensive load associated with aging and the decrease in the functional capacity [17], and broadening the opportunities for healthy aging. In this regard, it is possible to widely verify the use of several strategies of physical exercise to control SAH in patients in the literature [18,19] and improve the functional state of older adults [20,21,22].

The combination of antihypertensive drugs with physical exercise can control the BP and avoid the functional compromise of older adults [22]. Therefore, involving older adults in regular physical exercise that can stimulate the neuromuscular system minimizes the functional decrease related to aging, thus contributing to a more independent and healthier life [23].

The American School of Sports Medicine recommends a weekly combination of different types of training (cardiorespiratory, flexibility, force, and neuromotor training) [24]. Among the types of training that can provide a combination of these types of physical exercise, FT is highlighted as it promotes the improvement of physical capacities in an integrated, synergic, and balanced way, aiming at improving individual functionality. Thus, FT guarantees security and efficiency in performing daily life activities (DLAs), labor, and even sports activities [25].

Based on the content above, it is interesting to point out that, in Brazil, around 59% of public expenditures are related to SAH, representing more than BRL 2 billion per year [26], which entails high costs of public spending related to hospitalization and pharmacological treatments. It is essential to highlight that resistant arterial hypertension is related to cardiovascular events, presenting a 47% higher risk and the elevation of morbimortality when compared to hypertensive cases in general [27].

In this context, the following question is raised: Would FT be able to promote a chronic hypotensive effect and yet improve the physical fitness of older adults with resistant arterial hypertension? Although studies about physical training indicate promising results in improving life quality for RH patients [28], more specific recommendations about the chronic hypotensive effect and the functional state of treating resistant arterial hypertension still need to be made [29]. Therefore, it is essential to understand the responses of FT in the chronic hypotensive effect and the physical fitness of older adults with this clinical condition. Thus, this work aimed to assess the impact of functional training on ambulatory blood pressure after 24 sessions.

## 2. Materials and Methods

### 2.1. Characterization of the Research and Description of Subject Selection

This is a randomized and controlled clinical rehearsal, carried out by the CONSORT guidelines, held with 15 resistant hypertensive older adults assisted at the Health Basic Unit (HBU) in the Lagoa Seca neighborhood in the city of Juazeiro do Norte–CE, from September to November 2022. The sample size was determined based on the results of a previous study [30], and a possible 20% loss was added.

Then, the sample was allocated into two denominated groups: the control group (CG), consisting of seven resistant hypertensive older adults, and the experimental group (EG), consisting of eight resistant older adults. For the group allocation, we considered the stratified randomization method through Microsoft Excel 2010, considering the higher and lower BP values and executing the random function. The randomization was carried out by a researcher who was not involved in the recruitment or intervention of the participants.

### 2.2. Eligibility Criteria

For the current research, we included RH older adults who were 60 years old or more, sedentary or out of physical training in the last six months, who were using three or more antihypertensive agents in adequate doses and combinations, and who showed adherence to the treatment.

### 2.3. Exclusion Criteria

RH older adults with uncontrolled heart failure, Parkinson’s, Alzheimer’s, dementia, physical and visual disabilities, morbid obesity (Body Mass Index BMI ≥ 40 kg/m^2^), cancer, unstable angina, or osteoarticular dysfunctions that limit the practice of exercises were excluded. Those who did not fulfill 90% of the sessions and did not go through the ABPM exam were excluded.

### 2.4. Instruments and Procedures

#### 2.4.1. Anamnesis and Sociodemographic, Socioeconomic, and Clinical Characterization

For identifying general characteristics of HR older adults, an anamnesis and the application of a structured questionnaire were held by the researcher with questions about the sociodemographic (gender, age, education, marital status), socioeconomic (income), and clinical (level of physical activity, Parkinson’s, Alzheimer’s, dementia, motor disability, visual disability, cancer, unstable angina, osteoarticular dysfunctions, and body mass index) aspects.

To identify the criterion of sedentarism, we applied the Physical Activity International Questionnaire (IPAQ) in the short version [31], which aims to verify the physical activity level of several populations and sociocultural contexts. The short version of IPAQ has eight questions, making it an instrument of easy application, stability, and precision. The IPAQ classifications are the following: very active, active, irregularly active A, irregularly active B, and sedentary.

In the search for attending to the criterion of adherence to the medicine, we applied the therapy adherence scale of chronicle diseases composed of 8 items, which makes it possible to determine the level of adherence as high adherence (8 points), average adherence (6 or 7 points), and low adherence (<6 points) [32].

For attending to the criteria of morbid obesity (BMI ≥ 40), the calculation of the body mass index (BMI = kg/m^2^) was used. An electronic digital balance with a capacity of 150 kg was used for this purpose, with a precision of 50 g; Sanny’s and height were measured with the support of a 1.5 m inextensible measuring tape fixed perpendicularly to a plain wall, 1 m above the floor, without a baseboard.

#### 2.4.2. Evaluation of Ambulatorial Arterial Hypertension

BP was measured through the ABPM method, which is an indirect method of BP measurement for a period of 24 h or more while the user conducts their usual daily activities.

The ABPM (Contec, ABPM50) was programmed to measure every 20 min during the waking time and every 30 min during the sleeping time, obtaining a minimum of 16 measurements in the waking period and eight measurements in the sleeping period. These older adults were directed to follow all the steps in the Arterial Hypertension Brazilian Guidelines [6]. The mapping was always put on in the morning, around 10–11 a.m., and it was removed after 24 h. The daytime and nighttime periods were determined based on each participant’s self-report about their bedtime and wake-up time. In the post-test, the ABPM was entered 48 h after the latest experimental session.

#### 2.4.3. Evaluation of the Physical Fitness

For the physical fitness assessment, we used the Senior Fitness Test (SFT), composed of the following assessments: getting up from and sitting down on a chair (force and resistance of lower limbs); forearm flexion (force and resistance of upper limbs); sitting and reaching (flexibility of lower limbs); sitting, walking 2.44 meters, and sitting again (physical mobility, speed, agility, and dynamic balance); reaching behind the back (flexibility of upper limbs (shoulder); walking for 6 min (aerobic resistance). For classification, we summed up all of the scores of the General Physical Fitness Index (GPFI) of each test set, resulting in a total GPFI [33].

#### 2.4.4. Study Design

**Figure 1:**
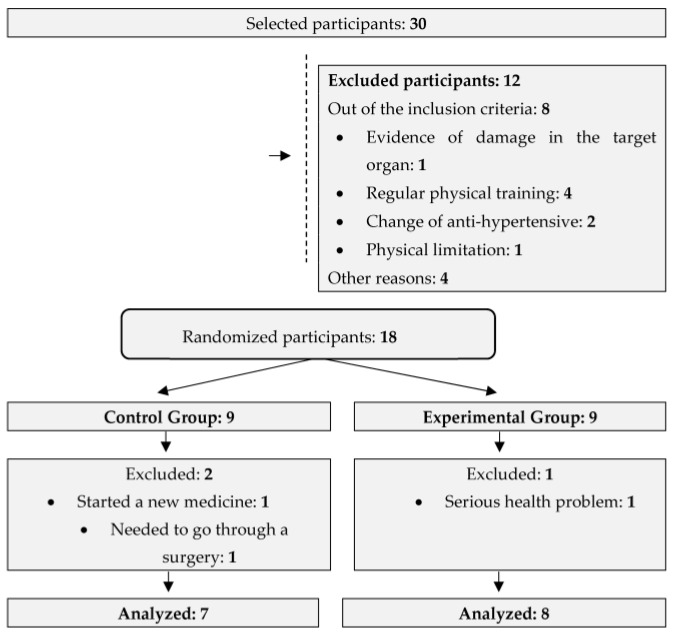
Flow chart to describe the study design.

The randomized clinical rehearsal was held with the aim of analyzing the effect of the functional training on the variable of resistant hypertension, describing the mentioned variables through the intervention of 24 sessions. After selecting the sample, the older adults were randomized into two groups: the control group (CG) and the experimental group (EG). Then, the initial assessment was held, which comprised the application of the anamnesis and conducting the sociodemographic, socioeconomic, and clinical characterization of the older adults, as shown in Figure 1

The intervention was held over 8 weeks of training with 3 weeks of sessions from 10 to 11 a.m., generating 24 training sessions, all of which lasted 60 min, with the intensity of each session measured through the OMNI-GSE scale [34] and subdivided into four blocks, as proposed in a previous study [25].

Block 1 was the mobility and preparation block (10 min and intensity OMNI-GSE: 2 to 3); 1 set of 15 s was performed for the exercises of mobility, while 2 sets of 20 s with 30 s of rest were performed for the exercises of preparation for the movement. Block 2 was the neuromuscular 1 block (20 min and intensity OMNI-GSE: 3 to 5). The exercises proposed in this block were performed in 3 sets of 30 s with 30 s of rest between the sets. Block 3 was the neuromuscular 2 block (25 min and intensity OMNI-GSE: 4 to 7). The exercises in this block were performed in 3 sets of 30 s each with 30 s of rest between the sets. Block 4 was the cardiometabolic block (duration of 5 min and intensity OMNI-GSE: 8 to 9). This block involved 8 sets of squatting for 15 s per 15 s of rest (from the 1st to 12th sessions) and 8 sets of squats for 30 s per 20 s of rest (13th to 24th session); the exercises of each block were divided by Table 1.

In order to familiarize the older adults with resistant hypertension with the intervention and with the scale of perception, familiarization with the processes of the exercises was conducted for two weeks. Then, the intervention of the 24 sessions was applied. We highlight that the CG did not undertake any training, and the researcher was responsible for keeping in touch weekly to confirm the information about the medicines (if there were any changes) as well as to assess whether the participants were conducting physical exercises during the research time.

### 2.5. Data Analysis

The treatment for the data analysis was elaborated on using a database in Microsoft Excel^®^ software, 2013. Then, the data analyses were conducted using JAMOVI, version (2.3.19). In the current study, the descriptive analyses were conducted using frequencies (absolute and percentages), in addition to the measures of central tendency and dispersion (rate and pattern deviation). To verify the data’s normality, homogeneity, and sphericity, we used the Shapiro–Wilk, Levene, and Mauchly’s tests, respectively. The association between the class of medicine and the allocation group was tested using Fisher’s exact test. An independent T-test was used to verify possible differences in the pre-intervention moment between the CG and the EG to demonstrate the equivalence between the groups.

An ANOVA of two factors for repeated measures was applied to verify the effect of interaction between time and group. Eta Squared was used to verify the size of the intervention effect. In all the analyses, we adopted an alpha of 0.05. In addition, the delta percentage calculation (Δ% = (pre-post) / absolute value (post) was used for the variations in the physical fitness tests.

### 2.6. Ethical Aspects

All the individuals gave their informed consent for the inclusion before participating in the study. The Declaration of Helsinki carried out the study. It was approved by the Ethical Committee of the University Center Dr. Leão Sampaio through statement number 5.611.163 and by the Brazilian Register of Clinical Rehearsals (RBR-867mysf).

## 3. Results

A total of 15 RH older adults participated in the research, and the majority of the seniors were females (13; 86.7%) with an average age of 70.1 ± 6.30 years. We observed that most have a family income higher than one minimum wage (10; 66.3%) and are illiterate (12; 80%). In the inferential analysis, we observed statistically significant differences between the groups in the weight and height variables (*p* > 0.05) (Table 2).

The ANOVA highlighted significant reductions in the variables in the average of 24 h for repeated measures (systolic: *p* = 0.003; diastolic: *p* = 0.004), daytime shift (systolic: *p* = 0.004; diastolic: *p* = 0.003) and night shift (systolic: *p* = 0.025: diastolic: *p* = 0.016). Nonetheless, the post hoc test pointed out that the difference in the DAP is significant only in the EG between the pre- and post-intervention periods, and differences between the groups do not occur in the post-intervention period.

We observed, however, that there were reductions in the 24-h average (∆) of 12 mmHg in the SAP and 6.9 mmHg in the DAP in EG. It is essential to highlight that the FT promoted a large effect size in all the studied variables (Table 3).

**Figure 2:**
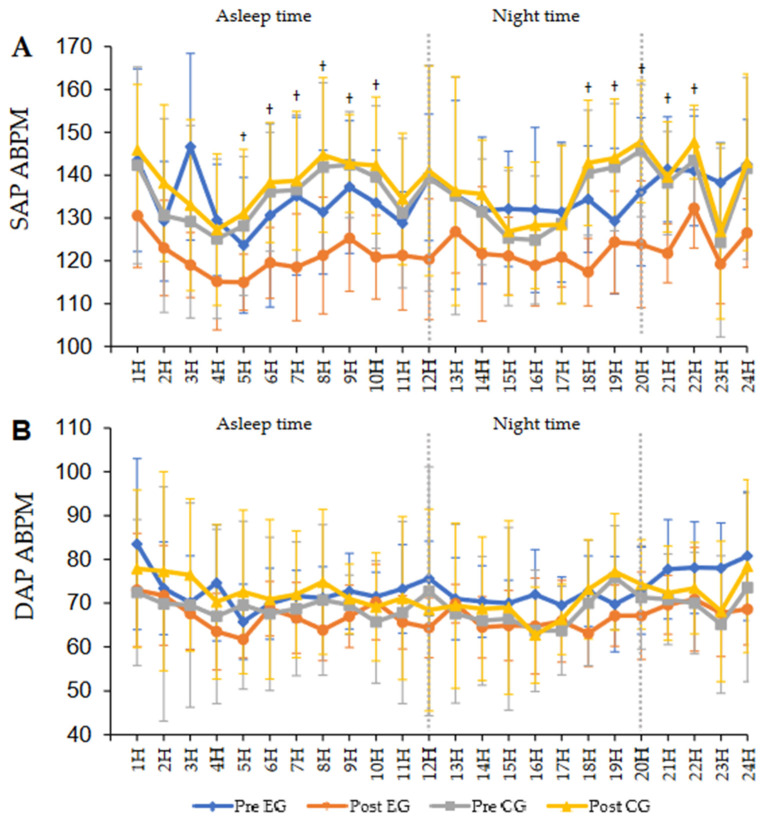
Average values of the behavior of the SAP and DAP along the 24 h of the ambulatorial mapping between the pre- and post-intervention periods between groups. Legend: ABPM—ambulatory blood pressure mapping; SAP—systolic arterial pressure; DAP—diastolic arterial pressure. ANOVA of repeated measures. † indicates significant difference *p* > 0.05.

Regarding the 24-h ABPM, Figure 2 presents the SAP and DAP average values per hour in the pre- and post-intervention periods and the interaction between the groups in 24 h. It is essential to highlight that the EG presented a reduction in both SAP (Figure 2A) and DAP (Figure 2B) throughout the 24 h of the exam, between the averages of the pre- and post-intervention periods. Regarding the CG, in the post-intervention period, both to the SAP (Figure 2A) and to the DAP (Figure 2B), increased values were observed during the 24 h of ABPM in the post-intervention period.

By assessing the post hoc results, we observed statistically significant differences between the groups and between the 24 h of ABPM in the post-intervention period, under the following systolic arterial pressures: 5 h (*p* = 0.025); 6 h (*p* = 0.010); 7 h (*p* = 0.025); 8 h (*p* = 0.020); 9 h (*p* = 0.021); 10 h (*p* = 0.011); 18 h (*p* = 0.002); 19 h (*p* = 0.016); 20 h (*p* = 0.011); 21 h (*p* = 0.007); 22 h (*p* = 0.008).

Table 4 presents the data on the general physical fitness of elderly people with RH. At the beginning of the intervention, the physical capabilities of force/resistance and flexibility (upper and lower limbs) and aerobic resistance were similar between the groups. Additionally, significant moderate effects were found after 24 FT sessions in the force/resistance variables of upper and lower limbs, physical mobility, and aerobic resistance.

The results obtained in Table 4 point to a significant improvement in the force and resistance variables of the lower and upper limbs, physical mobility, and aerobic resistance when the pre- and post-intervention periods are compared between GC and EG, which does not occur in the flexibility of lower and upper limbs. It is possible to highlight that, in EG, there was a percentage increase (Δ%) in all variables, highlighting the force and resistance of lower limbs (32.4%) and the force/resistance of upper limbs (72.7%).

The post hoc of Bonferroni indicated an increase in force/resistance of lower (*p* < 0.001) and upper limbs (*p* = 0.006), as well as in the physical mobility (*p* = 0.005) and the aerobic resistance (*p* = 0.002) between the groups only in the post-intervention period.

**Figure 3:**
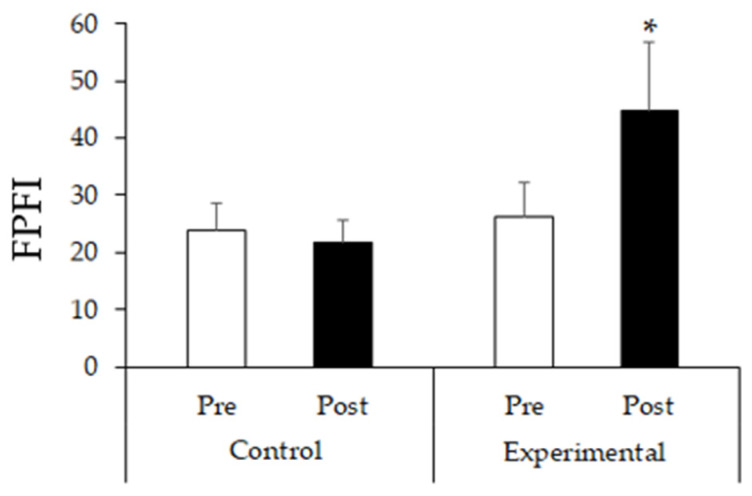
The data represent the average and a pattern deviation in the total FPFI stratified by the groups of older adults with resistant hypertension in the pre- and post-experimental phases of 24 functional training sessions. Abbreviations: FPFI—Functional Physical Fitness Index. ANOVA for repeated measures * *p* < 0.05.

Figure 3 demonstrates the effect of FT on the total FPFI, although it is possible to observe that the FT promoted an increase in this index. [f(1) = 13.78; *p* = 0.003]. The post hoc of Bonferroni indicated a difference only in the post-intervention period between the groups in the total FPFI (*p* < 0.001).

## 4. Discussion

The results highlight that FT caused a significant reduction in the SAP in older adults with elevated cardiovascular risk. We observed an average decrease of 12 mmHg in the BP during the 24 sessions and 6.9 mmHg in the DAP in the EG from the pre- to the post-treatment. Moreover, the results indicate a moderate effect of the intervention in all the variables of the ambulatorial arterial pressure.

The research demonstrated positive results regarding the increase in the total GPFI in the EG compared to the pre- and post-intervention periods concerning the CG. There also was a noticeable increase in the force/resistance in the upper and lower limbs, physical mobility (related to coordination), balance/agility, and aerobic resistance. In all these aspects, moderate effects of intervention were observed.

A study was conducted in Portugal [29] with people with resistant hypertension aged between 40 and 75 years old. Participants were divided into two groups: a group undertaking aerobic moderate exercises and a control group receiving habitual care. Their findings pointed to significant reductions in the average ABPM over 24 h of diurnal SAP (8 mmHg) and DAP (5.7 mmHg); these changes came after a period of 36 training sessions. No reductions occurred in the nighttime DAP.

A randomized study [35] concluded that the use of the combination of aerobic and resistance exercises in the same session evokes more prolonged reductions in the ambulatorial arterial pressure in RH, continuing for 12 h after the exercise in comparison to aerobic-only exercise (6 h) and resistance-only exercise (3 h). This can be justified by the mechanisms of action in which the type of exercise is involved for the control of BP; in an acute way, aerobic exercise seems to induce a reduction in ABPM in the daytime, while in the resistance exercises seem to bring more significant and longer reductions in RH during the night shift [35]. This result can be justified by the fact that aerobic exercise and resistance exercise work in different ways in the circadian cycle and release the possible control systems of the BP—for example, the autonomous modulation and neuro-humoral factors [36]. These can cause reductions in BP in different periods of the day in RH. However, it is essential to carry out significant investigations into this theory.

It is essential to highlight the fact that a reduction of 10 mmHg in the SAP or 4 mmHg in the DAP can represent a 30% decreased chance of cerebrovascular accident and a 20% decreased chance of myocardial infarction [37].

Thus, our study expands the knowledge about the chronic effects of physical exercise for a subgroup of clinically vulnerable patients with HAS.

A study [38] carried out with hypertensive older adults under pharmacological treatment evidenced that 12 weeks of multicomponent training divided into 2-week sessions provided improvements in the physical capabilities of participants; there were elevations (Δ%) observed in the force of the lower limbs (10%) and the upper limbs (12%), as well as improvements in aerobic capability (6%) and balance (6%). There was no observed improvements in flexibility (−2%); this is in line with the results of our study.

Improvements in aerobic capability can be related to improvements in cardiac output and increases in peripheral blood flow, induced by the effects of physical exercise. It is essential to highlight the fact that the attainment of a bad result in the 6-min walking test may indicate dysfunction in one or more systems—for example, in the cardiovascular, pulmonary, neuromuscular, or metabolic ones—which may reduce the possibility of conducting a DLA [39].

That said, the authors of [22] presented evidence that the multicomponent training associated with the antihypertensive medication—independent of therapy type—improves functional status among elderly people with hypertension. The inexistence of differences between the study groups [22], under the use of different medication classes, plus the practice of multicomponent exercise, may be associated with the protection of the target organs that are provoked by the exercise in the hypertension treatment.

It is essential to highlight that this study has limitations. For example, the size of the sample and the number of men involved in the research inhibit the detection of potential effects that could be found through comparisons between subgroups of men and women. In addition, the DAP values were already low in the pre-intervention period.

## 5. Conclusions

The results of our study show that the 24 FT sessions can reduce the systolic ambulatorial arterial pressure in RH for both the average of the 24 h BP and the day and night periods. Thus, physical training acts as a treatment for arterial hypertension. However, this type of training may positively influence general physical fitness, increasing the force/resistance of upper and lower limbs, physical mobility, and the aerobic resistance of older adults with resistant hypertension.

## Figures and Tables

**Table 1:** Division of the exercises by the block.

Block 1	
Exercise	Duration
Mobility of wrist with wood bar	1st to 24th session
Mobility of shoulder with elastic band	1st to 24th session
Mobility of hip—sitting on a bench, torso in forward bending, rotation in both directions	1st to 24th session
Squat with bench support	1st to 12th session
Squat without bench support	13th to 24th session
Block 2	
Exercise	Duration
Moving between cones in the same direction	1st to 12th session
Moving between cones in different directions	13th to 24th session
Throwing a medicine ball onto the ground	1st to 12th session
Throwing a medicine ball horizontally onto the wall	13th to 24th session
Going up and down on the platform step	1st to 12th session
Jumping onto the step	13th to 24th session
Coordination in the agility ladder (front movements of entering and getting out of the ladder)	1st to 12th session
Coordination in the agility ladder (lateral movements)	13th to 24th session
Vertical alternated waving with a battle rope (doing isometric squat at 45° degrees).	1st to 12th session
Vertical alternated waving with a battle rope (squat simultaneous to the rope movement)	13th to 24th session
Block 3	
Exercise	Duration
Dumbbell Thruster (with an elastic band resting on the feet)	1st to 12nd session
Dumbbell Thruster (with dumbbells)	13th to 24th session
Farmers walk (linearly walking with light weights)	1st to 12th session
Farmers walk (increasing the weight and passing through obstacles)	13th to 24th session
Deadlift with kettlebell (5 kg weight)	1st to 12nd session
Deadlift with kettlebell (8 kg weight)	13th to 24th session
Plank on the wall (performing the movement with the wall support)	1st to 12nd session
Plank on the wall (on a 40 cm bench)	13th to 24th session
Pelvic lift with pull over (pelvic elevation and pullover with light weights)	1st to 12th session
Pelvic lift with pull over (increase in the movement amplitude by putting the step support under the feet and increasing the load at the pullover)	13th to 24th session
Block 4	
Exercise	Duration
Eight sets of squats for 15 s per 15 s of rest	1st and 12th session
Eight sets of squats for 30 s per 20 s of rest	13th to 24th session

**Table 2:** Characterization of the resistant hypertensive older adults sample stratified by groups.

VARIABLES		Control (*n* = 7)	Experimental Group (*n* = 8)	*p*-Value
Anthropometrics Characteristics				
	Age (years)	72.9 ± 5.9	67.8 ± 5.9	0.786
	Weight (kg)	69.8 ± 8.8	78.5 ± 22.3	0.050 ^†^
	Height (m)	1.5 ± 0.0	1.6 ± 0.1	0.019 ^†^
	BMI (kg/m^2^)	28.3 ± 3.7	30.3 ± 5.4	0.463
Hemodynamics Parameters				
	SAP 24 h (mmHg)	134.0 ± 16.6	134.0 ± 10.8	0.990
	DAP 24 h (mmHg)	70.0 ± 16.0	74.4 ± 5.8	0.003 ^†^
	HR (bpm)	76.6 ± 7.0	71.9 ± 13.0	0.814
Use of Medication				
	Quantity (unit)	3.5 ± 0.5	3.2 ± 0.4	0.234
Class of Medicine				
	Diuretic	7 (100)	8 (100)	(-)
	ACEI	0	3 (37.5)	0.200
	Calcium channel inhibitors	7 (100)	8 (100)	(-)
	ARP	7 (100)	5 (62.5)	0.200
	Adrenergic Inhibitors	4 (57.1)	2 (25)	0.315

^†^ Independent *t*-test (adopted alpha *p* < 0.05). Abbreviations: BMI—body mass index; SAP—systolic arterial pressure; DAP—diastolic arterial pressure; HR—heart rate; ACEI—angiotensin-converting enzyme inhibitors; ARP—angiotensin receptor and blocker.

**Table 3:** Description and comparison of the arterial pressure and pressure load of the resistant hypertensive older adults, stratified by groups, after 24 sessions of functional working. Juazeiro do Norte, 2022.

Variables	Control Group				Experimental Group				*F*	*p*	*η* ^2^
	Pre		Post		Pre		Post				
Daytime SAP	135.0	17.1	137.0	15.6	131.0	9.8	123.0	7.7	11.79	0.004 *^†^	0.476
Daytime DAP	70.3	16.0	74.6	15.4	74.4	5.4	68.0	6.7	13.09	0.003 *	0.502
Night SAP	131.0	17.2	132.0	16.2	133.0	15.0	121.0	8.8	6.46	0.025 *^†^	0.332
Night DAP	65.1	14.5	68.3	11.7	72.0	8.6	65.3	6.5	7.73	0.016 *	0.373
Average 24 h SAP	134.0	16.6	135.0	15.0	134.0	10.8	122.0	7.5	13.60	0.003 *^†^	0.511
Average 24 h DAP	70.0	16.0	72.4	14.9	74.4	5.8	67.5	6.3	12.56	0.004 *	0.491

Abbreviation: SAP—systolic arterial pressure; DAP—diastolic arterial pressure. Values represent rate and pattern deviation. * ANOVA for repeated measures (significant differences between the pre- and post-intervention periods vs. groups *p* < 0.05). † Post hoc Bonferroni (difference between groups in the post-intervention periods).

**Table 4:** Description and comparison of resistant hypertensive older adults’ physical fitness stratified by groups, pre- and post-intervention, using 24 functional training sessions.

Tests		Control Group	Experimental Group	*p*	*η* ^2^
Force and resistance of lower limb (rep)					
	Pre	7.86 ± 2.34	10.8 ± 3.24	0.002	0.538
	Post	7.00 ± 1.53	14.3 ± 2.96 *^†^		
	Δ%	−10.8	32.4		
Force and resistance of upper limb (rep)					
	Pre	13.3 ± 4.46	12.8 ± 2.96	0.001	0.667
	Post	12.4 ± 4.16	22.1 ± 6.85 *^†^		
	Δ%	−6.8	72.7		
Flexibility of lower limbs (cm)					
	Pre	−19.8 ± 12.9	−10.0 ± 11.6	0.196	0.125
	Post	−21.9 ± 12.9	−8.0 ± 9.34		
	Δ%	−10.6	20.0		
Flexibility of upper limbs (cm)					
	Pre	−17.2 ± 7.25	−11.8 ± 14.7	0.779	0.006
	Post	−20.1 ± 6.62	−13.0 ± 6.83		
	Δ%	−16.2	−10.2		
Physical mobility (s)					
	Pre	10.2 ± 3.20	6.89 ± 1.19	0.003	0.494
	Post	12.3 ± 5.07	6.09 ± 1.52 *^†^		
	Δ%	−15.4	13.1		
Aerobic resistance (m)					
	Pre	327 ± 78.63	389 ± 62.8	0.001	0.608
	Post	309 ± 82.6	509 ± 115 *^†^		
	Δ%	−5.5	30.8		

Abbreviation: Values represent average and pattern deviation. * ANOVA for repeated measures, *p* < 0.05 for the pre- and post-intervention periods between groups. ^†^ Post hoc Bonferroni. Δ%: percentage variation.

## Data Availability

The raw data supporting the conclusions of this article will be made available by the authors upon request.
